# Flavonoids as therapeutic agents for epilepsy: unveiling anti-inflammatory and antioxidant pathways for novel treatments

**DOI:** 10.3389/fphar.2024.1457284

**Published:** 2024-09-12

**Authors:** Ya Zhang, Xizhuo Hu, Li-Qun Zou

**Affiliations:** ^1^ Department of Emergency Medicine, West China Hospital, Sichuan University/West China School of Nursing, Sichuan University, Chengdu, China; ^2^ School of Pharmacy, Chengdu University of Traditional Chinese Medicine, Chengdu, China

**Keywords:** flavonoids, epilepsy, neurological pathways, anti-inflammation, antioxidation

## Abstract

Epilepsy, a chronic neurological disorder affecting millions globally, is often exacerbated by neuroinflammation and oxidative stress. Existing antiepileptic drugs primarily manage symptoms, leaving the disease’s progression largely unaddressed. Flavonoids, ubiquitous plant metabolites with potent anti-inflammatory and antioxidant properties, show promise in epilepsy treatment. Unlike conventional therapies, they target multiple pathophysiological processes simultaneously, offering a comprehensive approach to this complex neurological disorder. This review explores the dual role of flavonoids in mitigating neuroinflammation and reducing oxidative stress through various molecular pathways. By inhibiting key inflammatory mediators and pathways such as NF-κB, MAPK, JNK, and JAK, flavonoids offer neuronal protection. They enhance the body’s natural antioxidant defenses by modulating enzyme activities, including superoxide dismutase, catalase, and glutathione peroxidase. Moreover, flavonoids influence crucial antioxidant response pathways like PI3K/AKT, Nrf2, JNK, and PKA. Despite their therapeutic promise, the low bioavailability of flavonoids poses a considerable challenge. However, cutting-edge strategies, including nanotechnology and chemical modifications, are underway to improve their bioavailability and therapeutic efficacy. These advancements support the potential of flavonoids as a valuable addition to epilepsy treatment strategies.

## Graphical Abstract

**Figure:**
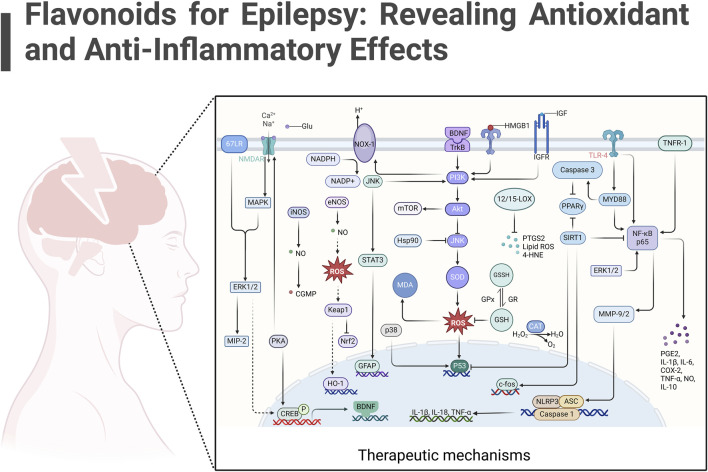
This review explores the potential of flavonoids in epilepsy treatment, emphasizing their anti-inflammatory and antioxidant mechanisms as potential therapeutic strategies.

## 1 Introduction

In 2005, epilepsy was identified as a chronic neurological disorder that predisposes individuals to seizures (Fisher et al., 2005). The World Health Organization (WHO) reports that over 70 million people worldwide are affected by epilepsy, with a higher prevalence in low- and middle-income nations (Thijs et al., 2019; Executive, 2020). Seizures stem from disrupted brain electrical activity due to an imbalance between excitatory and inhibitory neurotransmitters (Fisher et al., 2005; Fisher et al., 2014; Green et al., 2021). Neuroinflammation and oxidative stress caused by seizures worsen neuronal damage, increase seizure frequency and severity, and negatively affect patients’ quality of life and prognosis. Therefore, addressing inflammation and oxidative stress is essential for improving epilepsy outcomes. While antiseizure medications (ASMs) help 70% of patients, they have not notably changed seizure control and prevention (Rabidas et al., 2023; Levi-Abayo et al., 2024; Steinhoff et al., 2024).

Plant-derived compounds, particularly flavonoids, hold significant promise in addressing the limitations of conventional medications due to their broad pharmacological profiles and established safety. These natural compounds exhibit minimal side effects and present lower risks for developing drug resistance. When administered alongside ASMs, flavonoids can attenuate adverse reactions and enhance overall health outcomes (Alam et al., 2014; Zhang et al., 2015; Xiao, 2017). Previous pharmacological studies have demonstrated that flavonoids possess anti-inflammatory, antioxidant, and neuroprotective properties (Procházková et al., 2011; Keddy et al., 2012; Chen et al., 2018; Pan et al., 2019; Dourado et al., 2020; Heimfarth et al., 2021; Al-Khayri et al., 2022; Madireddy and Madireddy, 2023; Rabidas et al., 2023). As a result, they are now emerging as promising drug candidates for the treatment of epilepsy.

**FIGURE 1:**
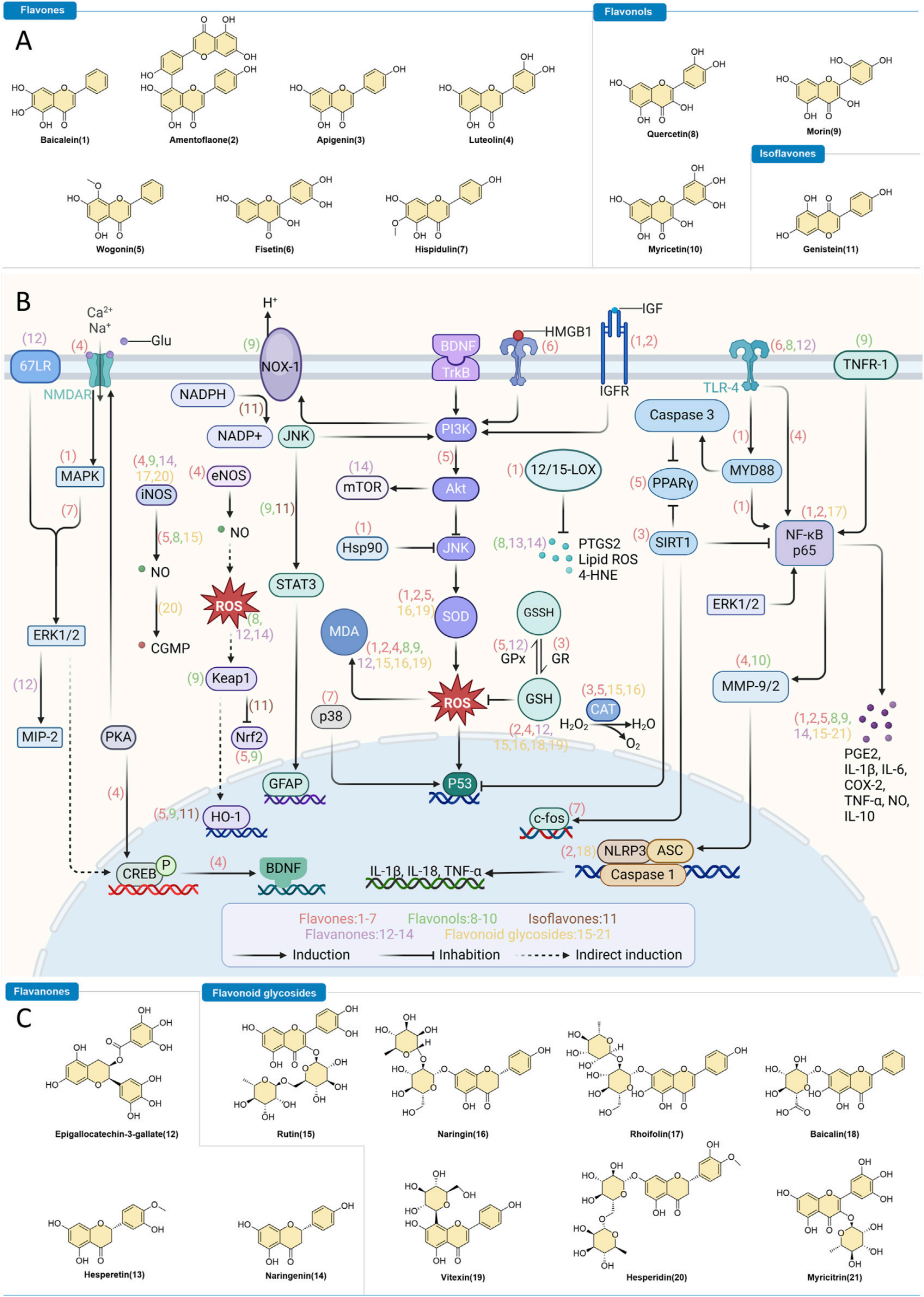
Flavonoids as Intervention Strategies for Epilepsy. **(A,C)** Chemical structures of flavonoids with regulatory effects on epilepsy. **(B)** Depiction of the therapeutic mechanisms through which bioactive flavonoids influence epilepsy treatment.

Despite the growing interest and significant findings in this field, there remains a lack of comprehensive, synthesizing review articles on flavonoids in epilepsy therapy. To address this gap, we conducted an extensive literature search using major databases such as Web of Science, PubMed, Scopus, and Google Scholar. We employed key terms including “flavonoids,” “epilepsy,” “neurological pathways,” “anti-inflammation,” and “antioxidation.” Our search was further enriched by relevant articles, bibliographic reviews, and co-author recommendations to ensure thorough coverage of the topic. This review aims to systematically summarize the advancements in research on plant-derived flavonoids in epilepsy treatment, providing a more detailed and nuanced understanding of this research trajectory. For ease of reference, we categorized flavonoids into two primary groups based on their pharmacological effects: anti-inflammatory and antioxidant, and elaborated on their potential mechanisms of action (Figure 1; Table 1).

**TABLE 1:** The detail mechanisms of flavonoids for the treatment of epilepsy.

Compound	Source	Detail mechanism	Cell lines/model	Dose	Application	Ref
Baicalein	The roots of *Scutellaria baicalensis* Georgi	Reduced the expression levels of IGF1R	Pilocarpine-induced epileptic rat model	20, 40, 80 mg/kg	*In Vivo*	Fu et al. (2020)
		Decreased IGF1R expression; lowered IL-1β, IL-6, and TNF-α levels	The microglial cell line BV2	5, 10 and 20 μM	*In Vitro*	Fu et al. (2020)
		Exhibited neuroprotective benefits against post-traumatic epileptic seizures by inhibiting ferroptosis	FeCl_3_-induced PTE model	50, 100 mg/kg	*In Vivo*	Li et al. (2019)
		Decreased ferroptotic indices (lipid ROS, 4-HNE, and PTGS 2) and inhibited the expression of 12/15-LOX	Ferric ammonium citrate-induced HT22 hippocampal neuron damage model	1, 2, 4, 8, 16 and 32 μM	*In Vitro*	Li et al. (2019)
		Reduced oxidative stress; inhibited inflammation; decreased levels of HSP70, phosphorylated JNK (pJNK), and phosphorylated p38 (p-p38), while increased phosphorylated ERK (p-ERK)	Ppilepsy-like tremor rat (TRM)	10, 20 and 40 mg/kg	*In Vivo*	Mao et al. (2014)
		Reduced the activity of MAO-B enzyme and alleviate reactive astrogliosis associated with inflammation	LPS-induced neuroinflammation mouse mode	5 mg/kg	*In Vivo*	Cho et al. (2024)
		Lowered levels of TNF-α, IL-6, and IL-1β; inhibited the activation of the TLR4/MyD88/NF-κB pathway	BCCAO established a VD rat model	50, 100 mg/kg	*In Vivo*	Song et al. (2024)
Amentoflavone	*Selaginella tamariscina*	Increased fluorescence intensity of NLRP3, ASC, and caspase-1; reduced IL-1β and IL-18 expression	LPS-induced BV2 microglial cells	10 μM	*In Vitro*	Rong et al. (2019)
		Suppressed NLRP3, leading to downregulation of IL-1β, IL-18, and TNF-α expression, as well as ASC and caspase-1 expression	PTZ-induced epileptic mice model	25 mg/kg	*In Vivo*	Rong et al. (2019)
		Reduced hippocampal NF-κB p65 activity; decreased MDA content and increased SOD and GSH levels	Pilocarpine-induced epileptic rat model	25 mg/kg	*In Vivo*	Li et al. (2021)
		Caused cell cycle arrest mainly at the G2/M phase; reduced cyclin B1 in the G2/M phase and elevated p27Kip1 in the G0/G1 phase	BV-2 cells	0.1, 1 and 5 μM	*In Vitro*	Liu et al. (2020)
Apigenin	*Apium Graveolens* L	Recovered the activity of AChE and DA, 5-HT and NE levels; suppressed oxidative stress, inhibited inflammation decreased the NO level	Adult male Wister albino rats	20 mg/kg	*In Vivo*	Albrakati (2023)
		Reduced neuronal loss and neurodegeneration; reduced the release of cytochrome	KA-induced rats model	50 mg/kg	*In Vivo*	Hashemi et al. (2019)
		Controlled the accumulation of MPO-generated HClO; relieved KA-induced ferroptosis	SH-SY5Y human neuroblastoma cells	20 μM	*In Vitro*	Shao et al. (2020)
		Controlled the KA-induced overexpression of HClO in the epileptic brains; downregulated of MPO levels and increased the expression of SIRT1 and GPx4	KA-induced BALB/c nude mice	60 mg/Kg	*In Vivo*	Shao et al. (2020)
Luteolin	*Reseda odorata* Linn	Increased CREB phosphorylation; upregulated the expression of BDNF	PTZ-induced rats model	50 or 100 mg/kg	*In Vivo*	Zhen et al. (2016)
		Restored microglia alterations and transiently boosting BDNF/TrkB signaling	*Cdkl5*+/−heterozygous female mice	10 mg/kg	*In Vivo*	Tassinari et al. (2022)
		Decreased microglia overactivation by inhibiting the N-Methyl-D-aspartate (NMDA) receptors	*Cdkl5* KO mice	10 mg/kg	*In Vivo*	Galvani et al. (2021)
		Reduced levels of pro-inflammatory cytokines TNF-α, IL-6, IL-1β, and increased the anti-inflammatory cytokine IL-10, inhibiting the TLR4/IκBα/NF-κB pathway activation	PTZ-induced rats model	10, 20, and 50 mg/kg	*In Vivo*	Cheng et al. (2024)
		Increased eNOS activity in the liver, kidney, and hippocampus; prevented the rise of iNOS activity in these tissues and restored eNOS and iNOS activity	PTZ-induced rats model	10 mg/kg	*In Vivo*	Birman et al. (2012)
Wogonin	*Scutellaria baicalensis* Georgi	Lowered the expression of TNF-α, IL-1β, and iNOS to reduce inflammatory and oxidative stress responses	The ICH mice mode	20 mg/kg	*In Vivo*	Zhuang et al. (2021)
		Inhibited caspase-3 proteins; increased the expression of phosphorylated Akt, Nrf2, and HO-1 via the PI3K pathway	The TBI rats model	40 mg/kg	*In Vivo*	Feng et al. (2022)
Fisetin	Vegetables and fruits	Reduced HMGB1 and TLR-4 levels; inhibited the release of inflammatory and apoptotic molecules	PTZ-induced mice model	5, 10, and 20 mg/kg	*In Vivo*	Khatoon et al. (2021)
		Increased the levels of lipid peroxidation and protein carbonyl through increasing the levels of antioxidants, Upregulated gene expressions of CREB and BDNF	PTZ-induced mice model	5, 10, and 20 mg/kg	*In Vivo*	Khatoon et al. (2023)
Hispidulin	*Salvia plebeia* R. Br	Reduced neuronal death in CA3; decreased c-fos expression and mitogen-activated protein kinases phosphorylation in the hippocampus	KA-induced rats model s	10 or 50 mg/kg	*In Vivo*	Lin et al. (2015)
Quercetin	The stem and leaves of *Fagopyrum esculentum* Moench	Lowered MDA levels and increased TAC levels in the prefrontal cortex; reduced gene expression of IL-1β, iNOS, NLRP3, and TNF-α	PTZ-induced mice model	10, 20, and 40 mg/kg	*In Vivo*	Tavakoli et al. (2023)
		Lowered iNOS, IL-6, TNF-α, MCP1, and IL-1β levels; downregulated hippocampal TLR-4/NF-κB signaling	Hypoxia-induced brain injury rats model	25, 50 and 100 mg/kg	*In Vivo*	Wu et al. (2022)
Morin	*Moraceae*	Downregulated the PGAM5/DRP-1 axis; decreased protein expression/content of TNFR-1, TNF-α, and IL-1β; inhibited the p-JAK2/p-STAT3 cascade and GFAP expression in the CA1 area	PTZ-induced rats model	10 mg/kg	*In Vivo*	Abd El-Aal et al. (2022)
Myricetin	The bark of *Morella rubra* Lour	Inhibited the BDNF-TrkB signaling pathway; induced the expression of Glutamic acid decarboxylase 65 and GABA_A_R; increased GABA levels while decreasing glutamate levels; dose-dependently downregulated MMP 9 activity	PTZ-induced mice model	100 or 200 mg/kg	*In Vivo*	Sun et al. (2019)
EpigallocatechIn-3-gallate	*Camellia sinensis*	Suppressed the NF-κB pathway after SE, reducing MCP-1 expression; eliminated CCR2-driven MIP-2 expression and decreased 67LR expression in astrocytes	Rats received a brain infusion kit 1 implant	50 µM	*In Vivo*	Kim et al. (2023)
		Increased the number of surviving pyramidal neurons; downregulated NF-κB, IL-1β, and TLR-4 expression	Lithium-pilocarpine TLE rats model	25 mg/kg	*In Vivo*	Qu et al. (2019)
Hesperetin	*Citrus reticulate* Blanco	Lowered p-4E-BP1 expression in the DG; reduced proinflammatory cytokines (TNF-α and IL-1β) and iNOS levels; suppressed GCD by inhibiting mTORC1 activation	KA-induced mice model	5, 10, and 20 mg/kg	*In Vivo*	Kwon et al. (2018)
Naringenin	*Citrus reticulata* Blanco	Deactivated mTORC1 in the hippocampus to inhibit GCD; reduced TNF-α and IL-1β expression in iba1-positive microglia in the DG	KA-induced mice model	50 and 100 mg/kg	*In Vivo*	Park et al. (2016)
		Scavenged intracellular ROS, reducing excess ROS and preventing stress-mediated apoptosis of cells	PTZ-induced zebrafish model	100 μM	*In Vivo*	Murugan et al. (2023)
Silibinin	*Silybum marianum* (L.) Gaertn	Reduced GCD by inhibiting mTORC1 in the DG; inhibited LC3B overexpression in hippocampal CA1 neurons; inhibited the increase in TNF-α and IL-1β levels	KA-induced mice model	50, 100, and 200 mg/kg	*In Vivo*	Kim et al. (2017)
Genistein	*Glycine max* (Linn.) Merr	Inhibited the JAK2/STAT3 signaling pathway; reduced the ratio of apoptotic proteins caspase-3 and Bax to anti-apoptotic protein Bcl-2	PTZ-induced rats model	5 and 15 mg/kg	*In Vivo*	Hu et al. (2021)
Rutin	*Sophora japonica* Linn	Decreased glutamate concentration and glutaminase expression in the hippocampus; reduced expression levels of IL-1β, IL-6, IL-1R1, TNF-α, HMGB1, and TLR-4	KA-induced rats model	50 and 100 mg/kg	*In Vivo*	Chang et al. (2022)
		Elevated BDNF levels, enhanced hippocampal activities of SOD and CAT enzymes, increased hippocampal Nrf2 and HO-1 levels, and reduced TNF-α, IL-6, and COX-2 levels	PTZ-induced mice model	100 mg/kg	*In Vivo*	Mohamed et al. (2023)
Naringin	*Citrus grandis* (L.) Osbeck	Decreased in LC3B expression in hippocampal CA1 neurons; reduced microglia activation; attenuated an increase in TNF-α within iba1-positive microglia	KA-induced mice model	80 mg/kg	*In Vivo*	Jeong et al. (2015)
Hesperidin	*Citrus reticulata* Blanco	Decreased MDA and nitrite; increased SOD, GSH, and catalase; restored mitochondrial enzyme activities	PTZ-induced mice model	100, 200 mg/kg	*In Vivo*	Kumar et al. (2013)
Baicalin	*Scutellaria baicalensis*	Downregulated Bax and increased Bcl-2 expression; decreased the levels of IL-1β and IL-6; decreased Caspase-1 expression levels	PTZ-induced rats model	100 mg/kg	*In Vivo*	Yang et al. (2021)
		Enhanced BDNF expression, promoted neurogenesis, and activated the P2RX7/NLRP3/IL-1β signaling pathway	PTZ-induced rats model	50 mg/kg	*In Vivo*	Yang et al. (2024)
Vitexin	The leaves of *Vitex negundo* L. var. cannabifolia	Decreased NKCC1 mRNA expression in peri-ischemic brain tissue; reduced hypoxia-ischemia-induced IL-1β, IL-6, neutrophil infiltration, and TNF expression	Rats underwent the ligation of the right common carotid artery	45 mg/kg	*In Vivo*	Luo et al. (2018)
		Prevented oxygen glucose deprivation-induced stress fiber formation in RBMECs via the NKCC1/F-actin pathway; restored ZO-1 expression and mitigated BBB breakdown	Rat brain microvascular endothelial cells	100 mM	*In Vitro*	Luo et al. (2018)
Myricitrin	*Canarium album* (Lour.) Raeusch	Improved passive avoidance memory; improved spatial learning and memory; improved hippocampal MDA, TAC, and TNF-α concentration	KA-induced rats model	5 mg/kg	*In Vivo*	Keikhaei et al. (2020)
Rhoifolin	*Rutaceae*	Dose-dependently restored SOD, MDA, GSH, and MPO levels; reduced TNF-α, IL-1β, and IL-6 levels; inhibited the NF-κB/iNOS/COX-2 axis	HT-22 cells with MgCl_2_ free medium	5, 10, and 20 uM	*In Vitro*	Qi and Liu (2022)

## 2 Role of neuroinflammation and oxidative stress in epilepsy

Epilepsy, a complex neurological disorder, is marked by recurrent seizures due to abnormal neuronal firing in the brain. Beyond electrical disturbances, its pathophysiology includes persistent neuroinflammation and elevated oxidative stress, which contribute to seizure activity and disease progression. Effective epilepsy management requires addressing these underlying mechanisms. Flavonoids have emerged as promising therapeutic candidates, offering the potential to target both neuroinflammation and oxidative stress simultaneously (de Melo et al., 2023; Li et al., 2023).

### 2.1 The interplay of neuroinflammation and oxidative stress in epilepsy

Neuroinflammation plays a crucial role in epilepsy progression. It involves the activation of microglia and astrocytes, resulting in the release of inflammatory mediators like cytokines, prostaglandins, and leukotrienes (Vezzani et al., 2019). This inflammatory response worsens neuronal damage, raises seizure frequency and severity, and creates a vicious cycle that further impairs neuronal function, contributing to the chronic nature of the disorder (Li et al., 2023). Oxidative stress is another pivotal player in epilepsy. Seizures cause excessive production of reactive oxygen species (ROS) and free radicals, overwhelming the brain’s antioxidant defenses. This oxidative stress results in lipid peroxidation, protein oxidation, and DNA damage, further injuring neurons and aggravating seizure activity (Geronzi et al., 2018). The cumulative effect of oxidative stress and neuroinflammation not only damages neurons but also impairs synaptic function and plasticity, which are vital for cognitive processes (Fabisiak and Patel, 2022).

### 2.2 The key impact of oxidative stress in neuronal damage during epilepsy

Owing to the key roles of neuroinflammation and oxidative stress in epilepsy, targeting these pathways presents a promising therapeutic approach. Anti-inflammatory and antioxidant strategies may disrupt the cycle of neuronal damage and seizure worsening, thus enhancing patient outcomes (Terrone et al., 2020). Flavonoids are natural compounds present in various fruits, vegetables, and other plants, possess strong anti-inflammatory and antioxidant properties. These properties make flavonoids particularly beneficial in the context of epilepsy. Flavonoids can reduce neuroinflammation by inhibiting the synthesis and release of inflammatory mediators. They also disrupt key inflammatory signaling pathways like NF-κB, MAPK, and JAK, which are crucial for regulating the inflammatory response (Al-Khayri et al., 2022). In addition to their anti-inflammatory effects, flavonoids are potent antioxidants. They can reduce the production of ROS and free radicals, enhance endogenous antioxidant defenses, and protect neurons from oxidative damage. By neutralizing harmful molecules and modulating the activity of antioxidant enzymes like superoxide dismutase, catalase, and glutathione peroxidase (Catarino et al., 2015), flavonoids help maintain neuronal health and function.

After briefly explaining the crucial roles of neuroinflammation and oxidative stress in epilepsy, we will now focus on how flavonoids exert their therapeutic effects by targeting these pathological processes in the next section.

## 3 Harnessing nature’s arsenal: flavonoid as novel therapeutic agents for epilepsy

### 3.1 Molecular mechanisms: how flavonoids counteract neuroinflammation in epilepsy

Flavonoids provide anti-inflammatory effects and protect nerve cells through various pathways. They inhibit the synthesis and release of inflammatory mediators such as prostaglandins, leukotrienes, and cytokines (e.g., TNF-α, IL-1β). By reducing these inflammatory mediators, flavonoids mitigate neuroinflammation and protect neurons from damage. Additionally, flavonoids disrupt multiple inflammatory signaling pathways, including NF-κB, MAPK, and JAK, which are crucial in regulating the inflammatory response.

Activation of Toll-like receptor (TLR) pathways can trigger microglia to produce inflammatory cytokines. Studies show that epigallocatechin gallate (EGCG), quercetin, fisetin, and luteolin significantly lower TLR-4/NF-κB and IL-1β levels in hippocampal neurons of a pilocarpine-induced mouse epilepsy model (Qu et al., 2019; Khatoon et al., 2021; Wu et al., 2022; Cheng et al., 2024). Furthermore, amentoflavone, rhoifolin, and baicalein curb inflammation by inhibiting NF-κB p65 signal transduction (Li et al., 2021; Qi and Liu, 2022; Cho et al., 2024). Baicalein also inhibits the TLR4/MyD88/NF-κB pathway, ameliorates hippocampal inflammation, and regulates gut flora (Song et al., 2024). Additionally, baicalin’s anticonvulsant effects may stem from its impact on the TLR-4/MYD88/Caspase-3 pathway (Yang et al., 2021). Wogonin alleviates neuroinflammation via the PPAR-γ pathway (Zhuang et al., 2021). Interestingly, nearly all these flavonoids reduce inflammatory mediators such as tumor necrosis factor-alpha (TNF-α), nitric oxide (NO), prostaglandin E2 (PGE2), interleukin-1 beta (IL-1β), interleukin-18 (IL-18), and interleukin-6 (IL-6) (Jeong et al., 2015; Park et al., 2016; Kim et al., 2017; Kwon et al., 2018; Luo et al., 2018; Keikhaei et al., 2020). Meanwhile, baicalein and rutin increase anti-inflammatory interleukin-10 (IL-10) levels, thereby mitigating epilepsy (Chang et al., 2022; Cho et al., 2024). Additionally, luteolin reduces seizure frequency by inhibiting MMP2 and MMP9, essential for controlling inflammation (Birman et al., 2012). Myricetin downregulates MMP-9, contributing to its antiepileptic effects (Sun et al., 2019), and morin addresses the inflammatory processes that mediate epileptogenesis by hindering the release of neuroinflammatory mediators such as tumor necrosis factor receptor 1 (TNFR-1) (Abd El-Aal et al., 2022).

In cases of CDD with early-onset epilepsy, luteolin slows disease progression by suppressing microglial responses, reducing NMDA receptor-induced cell death, and increasing brain-derived neurotrophic factor (BDNF) levels. These combined actions limit neuroinflammatory overactivation and provide neuroprotection (Galvani et al., 2021; Tassinari et al., 2022). Fisetin not only raises BDNF expression but also boosts cAMP-responsive element-binding protein (CREB) levels, thereby enhancing neuronal plasticity, learning, and memory (Khatoon et al., 2023). Additionally, baicalein has been demonstrated to significantly reduce cognitive dysfunction following epileptic seizures by mitigating inflammatory responses and targeting mitogen-activated protein kinase (MAPK) signaling pathways (Mao et al., 2014). Like baicalin, hispidulin also has the capability to regulate the MAPK signaling pathway. Hispidulin is effective in inhibiting c-fos and major MAPKs (ERK1/2, p38, JNK), thereby reducing microglial activation and the production of pro-inflammatory cytokines, consequently diminishing seizures and hippocampal inflammation (Lin et al., 2015). Status epilepticus (SE) triggers leukocyte infiltration and neuroinflammation via MCP-1 and MIP-2, mechanisms driven by microglia and astrocytes. Epigallocatechin gallate (EGCG) modulates these effects through the 67LR-ERK1/2-MIP-2 signaling cascade, thereby decreasing epilepsy-induced inflammation (Kim et al., 2023). During seizures, the JAK2/STAT3 pathway is activated, resulting in increased inflammation. Genistein and morin suppress JAK2/STAT3 signaling, with morin also reducing GFAP expression, thereby mitigating inflammation (Hu et al., 2021; Abd El-Aal et al., 2022).

Beyond the aforementioned signaling pathways, flavonoids play a significant role in epilepsy treatment by modulating enzyme activities and influencing other signaling pathways. Luteolin regulates nitric oxide (NO) production by decreasing inducible nitric oxide synthase (iNOS) activity and increasing endothelial nitric oxide synthase (eNOS) activity, thereby reducing neuroinflammation and alleviating seizures (Birman et al., 2012). Additionally, fisetin lowers the release of high-mobility group box 1 (HMGB1), reducing inflammation and neuronal damage in epilepsy (Khatoon et al., 2021). Baicalein specifically targets the insulin-like growth factor 1 receptor (IGF1R) in hippocampal tissue, alleviating inflammation and epileptogenic symptoms (Fu et al., 2020). Moreover, amentoflavone modulates gene expression in BV-2 microglial inflammation models, impacting cell cycle, apoptosis, and autophagy, thereby offering neuroprotection (Liu et al., 2020). Naringenin suppresses mTORC1 expression in hippocampal dentate gyrus (DG) neurons and astrocytes following kainic acid (KA) exposure, thus reducing mTORC1 activation (Park et al., 2016). Amentoflavone and baicalin also inhibit the activation of the NLRP3 inflammasome, a key inflammatory mediator (Rong et al., 2019; Yang et al., 2024). Baicalein and baicalin can also reduce the activity of monoamine oxidase B (MAO-B) enzyme, thereby alleviating reactive astrogliosis associated with inflammation (Cho et al., 2024).

Overall, flavonoids alleviate epilepsy-related neuroinflammation by modulating crucial signaling pathways, suppressing inflammatory mediators, and regulating glial cell activation. These actions collectively help protect neurons from damage. Specifically, flavones primarily counter epilepsy through interactions with pathways such as NF-κB p65, the TLR4/MyD88/NF-κB axis, PPAR-γ, MAPK, HMGB1, IGF1R, NLRP3, and MAO-B, which are essential in mitigating neuroinflammation. Flavonols exert therapeutic effects by targeting the TLR-4/NF-κB, TNFR-1, and JAK2/STAT3 pathways. Isoflavones specifically focus on the JAK2/STAT3 signaling pathway. Flavanones influence both the TLR-4/NF-κB and 67LR-ERK1/2-MIP-2 pathways. Flavonoid glycosides affect NLRP3 and MAO-B levels and regulate the MMP-9, NF-κB p65, and TLR-4/MYD88/Caspase-3 pathways. Despite these advancements, the clinical application of flavonoids is hindered by their low bioavailability. Future research should aim to address these limitations by enhancing bioavailability through nanotechnology and chemical modifications, exploring multi-target therapies, and investigating the potential of flavonoids in personalized medicine and as antiviral treatments for epilepsy.

### 3.2 Flavonoids as neuroprotective antioxidants: mitigating oxidative damage in epileptic conditions

Flavonoids have exhibited profound antioxidant effects in alleviating epilepsy symptoms. These bioactive compounds not only reduce the production of free radicals, reactive oxygen species (ROS), and lipid peroxides but also bolster endogenous antioxidant defenses. Flavonoids alleviate oxidative stress and subsequent inflammatory neuronal damage by neutralizing harmful molecules and regulating key antioxidant enzymes like superoxide dismutase (SOD), catalase (CAT), and glutathione peroxidase (GPx). Additionally, they modulate key signaling pathways like Nrf2, PKA, and PI3K, thereby enhancing neuronal protection.

During seizures, the suppression of the Kelch-like ECH-associated protein 1/nuclear factor erythroid 2-related factor 2 (Keap1/Nrf2) signaling pathway exacerbates oxidative stress. Genistein reactivates the Keap1/Nrf2 pathway, thereby increasing the levels of antioxidant proteins like heme oxygenase 1 (HO-1) and NAD(P)H quinone dehydrogenase 1, which reduce oxidative damage (Hu et al., 2021). Wogonin further boosts Nrf2 and HO-1 expression in the hippocampus, providing significant neuroprotection (Feng et al., 2022). Morin enhances this pathway by lowering levels of malondialdehyde (MDA), NADPH oxidase 1 (NOX-1), and Keap-1 while upregulating HO-1 expression (Abd El-Aal et al., 2022). In addition, luteolin stimulates brain-derived neurotrophic factor (BDNF) expression via the PKA/CREB/BDNF pathway, protecting neurons from oxidative stress and enhancing cognitive function in pentylenetetrazole (PTZ)-induced epilepsy (Zhen et al., 2016). Wogonin reduces oxidative stress in temporal lobe epilepsy through the PI3K/AKT signaling pathway (Feng et al., 2022). Baicalein modulates MAPK pathways, resulting in decreased expression of heat shock protein 90 (Hsp90) and downregulation of phosphorylated c-Jun N-terminal kinase (p-JNK) (Mao et al., 2014).

Flavonoids effectively reduce oxidative stress by modulating enzyme activity, decreasing malondialdehyde (MDA) levels, and increasing levels of glutathione and key antioxidant enzymes such as superoxide dismutase, catalase, glutathione peroxidase, and glutathione reductase. This collective action contributes to improved cognitive function (Hashemi et al., 2019; Shao et al., 2020; Albrakati, 2023; Mohamed et al., 2023; Murugan et al., 2023). Specifically, quercetin enhances total antioxidant capacity (TAC) in the prefrontal cortex, while myricitrin boosts TAC in hippocampal tissues (Keikhaei et al., 2020; Tavakoli et al., 2023). Apigenin ameliorates oxidative stress and confers neuronal protection by activating silent information regulator 1 (SIRT1), reducing myeloperoxidase (MPO)-derived hypochlorous acid (HClO), and modulating GPx expression (Shao et al., 2020). Inflammatory conditions often lead to excessive nitric oxide (NO) production, inducing further oxidative stress. Rhoifolin mitigates this stress via the inducible nitric oxide synthase (iNOS) pathway (Qi and Liu, 2022). Baicalein reduces lipid peroxidation and prevents iron-dependent cell death (ferroptosis) in post-traumatic epilepsy by inhibiting 12/15-lipoxygenase (12/15-LOX) and decreasing lipid reactive oxygen species (Lipid ROS), 4-hydroxynonenal (4-HNE), and prostaglandin-endoperoxide synthase 2 (PTGS2) in HT22 cells (Li et al., 2019). Elevated NO levels disrupt mitochondrial respiratory chain functions, leading to oxidative stress and apoptosis. Research by Kumar et al. demonstrated that hesperidin reduces NO production via the NO-cGMP pathway, thereby providing neuroprotective effects (Kumar et al., 2013).

In short, flavones exhibit a multifaceted therapeutic impact on epilepsy by engaging with various signaling pathways, such as Nrf2, HO-1, PKA/CREB/BDNF, PI3K/AKT, MAPK, and SIRT1, which are vital for neutralizing oxidative stress. Flavonols, in particular, modulate the expression of Keap-1, HO-1, and NOX-1, highlighting their essential role in epilepsy management. Flavanones are distinguished by their action on the NO-cGMP signaling pathway, whereas isoflavones focus on the Keap1/Nrf2 axis. Additionally, flavonoid glycosides contribute to anti-inflammatory efforts via the iNOS pathway. Collectively, flavonoids mitigate oxidative damage in epilepsy through a variety of antioxidant mechanisms, including free radical scavenging, enhancing endogenous defenses, regulating antioxidant enzymes, and preventing oxidative stress-induced conditions like ferroptosis. Future research should aim to enhance bioavailability and precision delivery, possibly through nanocarrier systems, and explore synergies with established antiepileptic medications and multi-target therapies. This could lead to more potent antioxidant approaches for a comprehensive epilepsy treatment regimen.

Next, we will briefly discuss and provide insights into future directions for flavonoid-based epilepsy therapies, exploring how these foundational research findings may be translated into clinical applications.

## 4 Conclusion and prospects: future directions in flavonoid-based epilepsy therapies

This review highlights the significant therapeutic potential of flavonoids in the treatment of epilepsy, emphasizing their anti-inflammatory and antioxidant properties. We present a comprehensive overview of the mechanisms through which flavonoids exert their beneficial effects. Involved in a wide range of complex biological processes, flavonoids have shown a remarkable capability to mitigate the neuroinflammation associated with epilepsy. They achieve this by fine-tuning key signaling pathways such as NF-κB, MAPK, and JAK/STAT3, which are central to the inflammatory process. Beyond their regulatory effects, flavonoids act as powerful antioxidants, effectively scavenging harmful free radicals that are hallmarks of oxidative stress in epilepsy. Furthermore, they initiate key pathways, including Keap1/Nrf2, PKA/CREB/BDNF, and PI3K/AKT, which enhance the activity of antioxidant enzymes and strengthen the body’s innate defenses. The multifaceted protective effects of flavonoids present a promising avenue for therapeutic intervention in epilepsy.

Despite their promise, the clinical effectiveness of flavonoids is often limited by their low bioavailability. However, innovative approaches, such as nanotechnology and chemical modification, have shown promise in significantly enhancing both the bioavailability and targeted delivery of these compounds. For example, Hashemian and colleagues developed quercetin-conjugated Fe_3_O_4_-β-cyclodextrin nanoparticles, which have demonstrated a substantial increase in bioavailability and antiepileptic potency (Hashemian et al., 2019). Similarly, Ahmad and his team engineered chitosan-coated poly (lactic-co-glycolic acid) (PLGA) nanoparticles capable of crossing the blood-brain barrier when administered intranasally (Ahmad et al., 2020). Copmans and colleagues discovered that methylation of naringenin improves its metabolic stability and permeability across biological membranes, thereby enhancing absorption and bioavailability (Copmans et al., 2018). Gupta and his research group developed naringin-loaded transniosomes, which have been proven to increase solubility, permeability, and bioavailability, allowing for efficient intranasal administration during seizures (Gupta et al., 2023). Despite these scientific advances, there is a significant lack of clinical trial data on the use of flavonoids in epilepsy treatment, which continues to impede their wider clinical adoption.

Looking ahead, future research should prioritize developing multi-targeted combination therapies involving flavonoids. These therapies have the potential to simultaneously modulate various inflammatory and oxidative stress signaling pathways, offering a comprehensive approach to controlling the complex pathomechanisms underlying epilepsy (Löscher and Klein, 2022; Park et al., 2023). Genetic factors have been identified as key contributors to epilepsy (Symonds et al., 2019). Integrating flavonoids with gene therapy could pave the way for individualized treatment plans tailored to specific genetic mutations responsible for epilepsy, allowing for precise therapeutic interventions (Street et al., 2023). Furthermore, the potential of flavonoids in antiviral therapy is noteworthy, given their ability to inhibit viral replication and attenuate virus-induced inflammation (Badshah et al., 2021). Inhibiting viral replication can reduce virus-induced neuroinflammation, thereby decreasing the frequency and severity of seizures (Barker-Haliski et al., 2024; Costa and Vale, 2024). Future research should continue to delve into the antiviral properties of flavonoids to uncover their full potential in epilepsy treatment. These endeavors are crucial for advancing our understanding of plant-derived compounds in epilepsy therapy and for enhancing their clinical applications.

## References

[B1] Abd El-Aal S. A. El-Abhar H. S. Abulfadl Y. S. (2022). Morin offsets PTZ-induced neuronal degeneration and cognitive decrements in rats: the modulation of TNF-α/TNFR-1/RIPK1,3/MLKL/PGAM5/Drp-1, IL-6/JAK2/STAT3/GFAP and Keap-1/Nrf-2/HO-1 trajectories. Eur. J. Pharmacol. 931, 175213. 10.1016/j.ejphar.2022.175213 35981604

[B2] Ahmad N. Ahmad R. Alrasheed R. A. Almatar H. M. A. Al-Ramadan A. S. Amir M. (2020). Quantification and evaluations of catechin hydrate polymeric nanoparticles used in brain targeting for the treatment of epilepsy. Pharmaceutics 12 (3), 203. 10.3390/pharmaceutics12030203 32120778 PMC7150881

[B3] Alam M. A. Subhan N. Rahman M. M. Uddin S. J. Reza H. M. Sarker S. D. (2014). Effect of citrus flavonoids, naringin and naringenin, on metabolic syndrome and their mechanisms of action. Adv. Nutr. 5 (4), 404–417. 10.3945/an.113.005603 25022990 PMC4085189

[B4] Albrakati A. (2023). Monosodium glutamate induces cortical oxidative, apoptotic, and inflammatory challenges in rats: the potential neuroprotective role of apigenin. Environ. Sci. Pollut. Res. Int. 30 (9), 24143–24153. 10.1007/s11356-022-23954-1 36334201

[B5] Al-Khayri J. M. Sahana G. R. Nagella P. Joseph B. V. Alessa F. M. Al-Mssallem M. Q. (2022). Flavonoids as potential anti-inflammatory molecules: a review. Molecules 27 (9), 2901. 10.3390/molecules27092901 35566252 PMC9100260

[B6] Badshah S. L. Faisal S. Muhammad A. Poulson B. G. Emwas A. H. Jaremko M. (2021). Antiviral activities of flavonoids. Biomed. Pharmacother. 140, 111596. 10.1016/j.biopha.2021.111596 34126315 PMC8192980

[B7] Barker-Haliski M. DePaula-Silva A. B. Pitsch J. Sontheimer H. Hirsch L. J. Galanopoulou A. S. (2024). Brain on fire: how brain infection and neuroinflammation drive worldwide epilepsy burden. Epilepsy. Curr. 10.1177/15357597241242238 PMC11562294 39554268

[B8] Birman H. Dar K. A. Kapucu A. Acar S. Uzüm G. (2012). Effects of luteolin on liver, kidney and brain in pentylentetrazol-induced seizures: involvement of metalloproteinases and NOS activities. Balk. Med. J. 29 (2), 188–196. 10.5152/balkanmedj.2011.030 PMC4115855 25206993

[B9] Catarino M. D. Alves-Silva J. M. Pereira O. R. Cardoso S. M. (2015). Antioxidant capacities of flavones and benefits in oxidative-stress related diseases. Curr. Top. Med. Chem. 15 (2), 105–119. 10.2174/1568026615666141209144506 25547095

[B10] Chang A. Chang Y. Wang S. J. (2022). Rutin prevents seizures in kainic acid-treated rats: evidence of glutamate levels, inflammation and neuronal loss modulation. Food. Funct. 13 (20), 10401–10414. 10.1039/d2fo01490d 36148811

[B11] Chen L. Teng H. Jia Z. Battino M. Miron A. Yu Z. (2018). Intracellular signaling pathways of inflammation modulated by dietary flavonoids: the most recent evidence. Crit. Rev. Food. Sci. Nutr. 58 (17), 2908–2924. 10.1080/10408398.2017.1345853 28682647

[B12] Cheng Y. H. Zhang Y. Y. Huang P. X. Cheng Q. Z. Ding H. (2024). Luteolin ameliorates pentetrazole-induced seizures through the inhibition of the TLR4/NF-κB signaling pathway. Epilepsy. Res. 201, 107321. 10.1016/j.eplepsyres.2024.107321 38382229

[B13] Cho J. Y. Hong E. B. Kim Y. S. Song J. B. Ju Y. H. Kim H. (2024). Baicalin and baicalein from *Scutellaria baicalensis* Georgi alleviate aberrant neuronal suppression mediated by GABA from reactive astrocytes. CNS. Neurosci. Ther. 30 (5), e14740. 10.1111/cns.14740 38715318 PMC11076983

[B14] Copmans D. Orellana-Paucar A. M. Steurs G. Zhang Y. Ny A. Foubert K. (2018). Methylated flavonoids as anti-seizure agents: naringenin 4',7-dimethyl ether attenuates epileptic seizures in zebrafish and mouse models. Neurochem. Int. 112, 124–133. 10.1016/j.neuint.2017.11.011 29174382

[B15] Costa B. Vale N. (2024). Virus-Induced Epilepsy vs. Epilepsy patients acquiring viral infection: unravelling the complex relationship for precision treatment. Int. J. Mol. Sci. 25 (7), 3730. 10.3390/ijms25073730 38612542 PMC11011490

[B16] de Melo A. D. Freire V. A. F. Diogo I. L. Santos H. D. Barbosa L. A. de Carvalho L. E. D. (2023). Antioxidant therapy reduces oxidative stress, restores Na,K-ATPase function and induces neuroprotection in rodent models of seizure and epilepsy: a systematic review and meta-analysis. Antioxidants 12 (7), 1397. 10.3390/antiox12071397 37507936 PMC10376594

[B17] Dourado N. S. Souza C. D. S. de Almeida M. M. A. Bispo da Silva A. Dos Santos B. L. Silva V. D. A. (2020). Neuroimmunomodulatory and neuroprotective effects of the flavonoid apigenin in *in vitro* models of neuroinflammation associated with alzheimer’s disease. Front. Aging. Neurosci. 12, 119. 10.3389/fnagi.2020.00119 32499693 PMC7243840

[B18] Executive B. (2020). Epilepsy: report by the director-general. Geneva: World Health Organization.

[B19] Fabisiak T. Patel M. (2022). Crosstalk between neuroinflammation and oxidative stress in epilepsy. Front. Cell. Dev. Biol. 10, 976953. 10.3389/fcell.2022.976953 36035987 PMC9399352

[B20] Feng Y. Ju Y. Yan Z. Ji M. Yang M. Wu Q. (2022). Protective role of wogonin following traumatic brain injury by reducing oxidative stress and apoptosis via the PI3K/Nrf2/HO-1 pathway. Int. J. Mol. Med. 49 (4), 53. 10.3892/ijmm.2022.5109 35179214 PMC8904077

[B21] Fisher R. S. Acevedo C. Arzimanoglou A. Bogacz A. Cross J. H. Elger C. E. (2014). ILAE official report: a practical clinical definition of epilepsy. Epilepsia 55 (4), 475–482. 10.1111/epi.12550 24730690

[B22] Fisher R. S. van Emde Boas W. Blume W. Elger C. Genton P. Lee P. (2005). Epileptic seizures and epilepsy: definitions proposed by the international league against epilepsy (ILAE) and the international bureau for epilepsy (IBE). Epilepsia 46 (4), 470–472. 10.1111/j.0013-9580.2005.66104.x 15816939

[B23] Fu P. Yuan Q. Sun Y. Wu X. Du Z. Li Z. (2020). Baicalein ameliorates epilepsy symptoms in a pilocarpine-induced rat model by regulation of IGF1R. Neurochem. Res. 45 (12), 3021–3033. 10.1007/s11064-020-03150-8 33095440

[B24] Galvani G. Mottolese N. Gennaccaro L. Loi M. Medici G. Tassinari M. (2021). Inhibition of microglia overactivation restores neuronal survival in a mouse model of CDKL5 deficiency disorder. J. Neuroinflammation 18 (1), 155. 10.1186/s12974-021-02204-0 34238328 PMC8265075

[B25] Geronzi U. Lotti F. Grosso S. (2018). Oxidative stress in epilepsy. Expert. Rev. Neurother. 18 (5), 427–434. 10.1080/14737175.2018.1465410 29651881

[B26] Green R. Abe C. Denney D. A. Zhang R. Doyle A. Gadelmola K. (2021). Physical activity status and quality of life in patients with epilepsy - survey from level four epilepsy monitoring units. Epilepsy. Res. 173, 106639. 10.1016/j.eplepsyres.2021.106639 33865047

[B27] Gupta I. Adin S. N. Aqil M. Mujeeb M. (2023). Nose to brain delivery of naringin loaded transniosomes for epilepsy: formulation, characterisation, blood-brain distribution and invivo pharmacodynamic evaluation. J. Liposome. Res. 34 (1), 60–76. 10.1080/08982104.2023.2214619 37212622

[B28] Hashemi P. Babaei J. F. Vazifekhah S. Nikbakht F. (2019). Evaluation of the neuroprotective, anticonvulsant, and cognition-improvement effects of apigenin in temporal lobe epilepsy: involvement of the mitochondrial apoptotic pathway. Iran. J. Basic. Med. Sci. 22 (7), 752–758. 10.22038/ijbms.2019.33892.8064 32373296 PMC7196342

[B29] Hashemian M. Ghasemi-Kasman M. Ghasemi S. Akbari A. Moalem-Banhangi M. Zare L. (2019). Fabrication and evaluation of novel quercetin-conjugated Fe_(3)_O_(4)_-β-cyclodextrin nanoparticles for potential use in epilepsy disorder. Int. J. Nanomedicine. 14, 6481–6495. 10.2147/IJN.S218317 31496698 PMC6698168

[B30] Heimfarth L. Nascimento L. S. Amazonas da Silva M. J. Lucca Junior W. Lima E. S. Quintans-Júnior L. J. (2021). Neuroprotective and anti-inflammatory effect of pectolinarigenin, a flavonoid from Amazonian Aegiphila integrifolia (Jacq.), against lipopolysaccharide-induced inflammation in astrocytes via NFκB and MAPK pathways. Food. Chem. Toxicol. 157, 112538. 10.1016/j.fct.2021.112538 34500010

[B31] Hu Q. P. Yan H. X. Peng F. Feng W. Chen F. F. Huang X. Y. (2021). Genistein protects epilepsy-induced brain injury through regulating the JAK2/STAT3 and Keap1/Nrf2 signaling pathways in the developing rats. Eur. J. Pharmacol. 912, 174620. 10.1016/j.ejphar.2021.174620 34752743

[B32] Jeong K. H. Jung U. J. Kim S. R. (2015). Naringin attenuates autophagic stress and neuroinflammation in kainic acid-treated Hippocampus *in vivo* . Altern. Med. 2015, 354326. 10.1155/2015/354326 PMC4466392 26124853

[B33] Keddy P. G. W. Dunlop K. Warford J. Samson M. L. Jones Q. R. D. Rupasinghe H. P. V. (2012). Neuroprotective and anti-inflammatory effects of the flavonoid-enriched fraction AF4 in a mouse model of hypoxic-ischemic brain injury. PloS. One. 7 (12), e51324. 10.1371/journal.pone.0051324 23251498 PMC3520852

[B34] Keikhaei F. Mirshekar M. A. Shahraki M. R. Dashipour A. (2020). Antiepileptogenic effect of myricitrin on spatial memory and learning in a kainate-induced model of temporal lobe epilepsy. Learn. Motiv. 69, 101610. 10.1016/j.lmot.2019.101610

[B35] Khatoon S. Agarwal N. B. Samim M. Alam O. (2021). Neuroprotective effect of fisetin through suppression of IL-1R/TLR axis and apoptosis in pentylenetetrazole-induced kindling in mice. Front. Neurol. 12, 689069. 10.3389/fneur.2021.689069 34354662 PMC8333701

[B36] Khatoon S. Samim M. Dahalia M. Nidhi N. (2023). Fisetin provides neuroprotection in pentylenetetrazole-induced cognition impairment by upregulating CREB/BDNF. Eur. J. Pharmacol. 944, 175583. 10.1016/j.ejphar.2023.175583 36764352

[B37] Kim J. E. Lee D. S. Kang T. C. (2023). Epigallocatechin-3-gallate attenuates leukocyte infiltration in 67-kDa laminin receptor-dependent and -independent pathways in the rat frontoparietal cortex following status epilepticus. Antioxidants (Basel) 12 (4), 969. 10.3390/antiox12040969 37107345 PMC10136333

[B38] Kim S. Jung U. J. Oh Y. S. Jeon M. T. Kim H. J. Shin W. H. (2017). Beneficial effects of silibinin against kainic acid-induced neurotoxicity in the hippocampus *in vivo* . Exp. Neurobiol. 26 (5), 266–277. 10.5607/en.2017.26.5.266 29093635 PMC5661059

[B39] Kumar A. Lalitha S. Mishra J. (2013). Possible nitric oxide mechanism in the protective effect of hesperidin against pentylenetetrazole (PTZ)-induced kindling and associated cognitive dysfunction in mice. Epilepsy. Behav. 29 (1), 103–111. 10.1016/j.yebeh.2013.06.007 23939034

[B40] Kwon J. Y. Jung U. J. Kim D. W. Kim S. Moon G. J. Hong J. (2018). Beneficial effects of hesperetin in a mouse model of temporal lobe epilepsy. J. Med. Food. 21 (12), 1306–1309. 10.1089/jmf.2018.4183 30136878

[B41] Levi-Abayo S. Ben-Shabat S. Gandelman-Marton R. (2024). Guidelines and epilepsy practice: antiseizure medication initiation following an unprovoked first seizure in adults. Epilepsy. Res. 200, 107304. 10.1016/j.eplepsyres.2024.107304 38237220

[B42] Li Q. Li Q. Q. Jia J. N. Sun Q. Y. Zhou H. H. Jin W. L. (2019). Baicalein exerts neuroprotective effects in FeCl(3)-induced posttraumatic epileptic seizures via suppressing ferroptosis. Front. Pharmacol. 10, 638. 10.3389/fphar.2019.00638 31231224 PMC6568039

[B43] Li W. J. Wu J. Z. Zeng Y. N. Zheng W. (2023). Neuroinflammation in epileptogenesis: from pathophysiology to therapeutic strategies. Front. Immunol. 14, 1269241. 10.3389/fimmu.2023.1269241 38187384 PMC10771847

[B44] Li X. Q. Liu J. Yang J. H. Lai Z. H. (2021). Effects of amentoflavone on hippocampal neuron damage and oxidative stress in drug-induced epileptic rats. Acta. Medica. Mediterr. 37 (4), 2151–2155. 10.19193/0393-6384_2021_4_336

[B45] Lin T. Y. Lu C. W. Wang S. J. Huang S. K. (2015). Protective effect of hispidulin on kainic acid-induced seizures and neurotoxicity in rats. Eur. J. Pharmacol. 755, 6–15. 10.1016/j.ejphar.2015.02.041 25746462

[B46] Liu Z. Wang F. Ma H. Xia H. Tian J. Sun T. (2020). Amentoflavone induces cell cycle arrest, apoptosis, and autophagy in BV-2 cells. Front. Biosci. Landmark Ed. 25 (5), 798–816. 10.2741/4835 31585918

[B47] Löscher W. Klein P. (2022). New approaches for developing multi-targeted drug combinations for disease modification of complex brain disorders. Does epilepsy prevention become a realistic goal? Pharmacol. Ther. 229, 107934. 10.1016/j.pharmthera.2021.107934 34216705

[B48] Luo W. Min J. Huang W.-X. Wang X. Peng Y. Han S. (2018). Vitexin reduces epilepsy after hypoxic ischemia in the neonatal brain via inhibition of NKCC1. J. Neuroinflammation. 15 (1), 186. 10.1186/s12974-018-1221-6 29925377 PMC6011387

[B49] Madireddy S. Madireddy S. (2023). Therapeutic strategies to ameliorate neuronal damage in epilepsy by regulating oxidative stress, mitochondrial dysfunction, and neuroinflammation. Brain. Sci. 13 (5), 784. 10.3390/brainsci13050784 37239256 PMC10216584

[B50] Mao X. Cao Y. Li X. Yin J. Wang Z. Zhang Y. (2014). Baicalein ameliorates cognitive deficits in epilepsy-like tremor rat. Neurol. Sci. 35 (8), 1261–1268. 10.1007/s10072-014-1695-7 24590842

[B51] Mohamed K. M. Abdelfattah M. S. El-khadragy M. Al-Megrin W. A. Fehaid A. Kassab R. B. (2023). Rutin-loaded selenium nanoparticles modulated the redox status, inflammatory, and apoptotic pathways associated with pentylenetetrazole-induced epilepsy in mice. Green. Process. Synth. 12 (1), 20230010. 10.1515/gps-2023-0010

[B52] Murugan R. Mukesh G. Haridevamuthu B. Priya P. S. Pachaiappan R. Almutairi B. O. (2023). “Plausible antioxidant and anticonvulsant potential of brain targeted naringenin-conjugated graphene oxide nanoparticles,” in Biomass conversion and biorefinery (Springer).

[B53] Pan R. Y. Ma J. Kong X. X. Wang X. F. Li S. S. Qi X. L. (2019). Sodium rutin ameliorates Alzheimer's disease-like pathology by enhancing microglial amyloid-β clearance. Sci. Adv. 5 (2), eaau6328. 10.1126/sciadv.aau6328 30820451 PMC6393001

[B54] Park J. Jeong K. H. Shin W. H. Bae Y. S. Jung U. J. Kim S. R. (2016). Naringenin ameliorates kainic acid-induced morphological alterations in the dentate gyrus in a mouse model of temporal lobe epilepsy. Neuroreport 27 (15), 1182–1189. 10.1097/WNR.0000000000000678 27584687

[B55] Park S. Kim M. Lin Y. X. Hong M. Nam G. Mieczkowski A. (2023). Designing multi-target-directed flavonoids: a strategic approach to Alzheimer's disease. Chem. Sci. 14 (35), 9293–9305. 10.1039/d3sc00752a 37712013 PMC10498667

[B56] Procházková D. Boušová I. Wilhelmová N. (2011). Antioxidant and prooxidant properties of flavonoids. Fitoterapia 82 (4), 513–523. 10.1016/j.fitote.2011.01.018 21277359

[B57] Qi H. Liu L. (2022). Rhoifolin attenuates damage to hippocampal neuronal culture model of acquired epilepsy *in vitro* by regulating NF-κB/iNOS/COX-2 axis. Qual. Assur. Saf. Crop. 14 (3), 116–123. 10.15586/qas.v14i3.1093

[B58] Qu Z. Z. Jia L. J. Xie T. Zhen J. L. Si P. P. Cui Z. Q. (2019). (-)-Epigallocatechin-3-gallate protects against lithium-pilocarpine-induced epilepsy by inhibiting the toll-like receptor 4 (TLR4)/Nuclear Factor-kappa B (NF-kappa B) signaling pathway. Med. Sci. Monit. 25, 1749–1758. 10.12659/MSM.915025 30843525 PMC6417148

[B59] Rabidas S. S. Prakash C. Tyagi J. Suryavanshi J. Kumar P. Bhattacharya J. (2023). A comprehensive review on anti-inflammatory response of flavonoids in experimentally-induced epileptic seizures. Brain. Sci. 13 (1), 102. 10.3390/brainsci13010102 36672083 PMC9856497

[B60] Rong S. Wan D. Fan Y. Liu S. Sun K. Huo J. (2019). Amentoflavone affects epileptogenesis and exerts neuroprotective effects by inhibiting NLRP3 inflammasome. Front. Pharmacol. 10, 856. 10.3389/fphar.2019.00856 31417409 PMC6682693

[B61] Shao C. Yuan J. Liu Y. Qin Y. Wang X. Gu J. (2020). Epileptic brain fluorescent imaging reveals apigenin can relieve the myeloperoxidase-mediated oxidative stress and inhibit ferroptosis. Proc. Natl. Acad. Sci. U. S. A. 117 (19), 10155–10164. 10.1073/pnas.1917946117 32327603 PMC7229752

[B62] Song J. X. Li M. X. Kang N. Jin W. Xiao Y. N. Li Z. (2024). Baicalein ameliorates cognitive impairment of vascular dementia rats via suppressing neuroinflammation and regulating intestinal microbiota. Brain. Res. Bull. 208, 110888. 10.1016/j.brainresbull.2024.110888 38295883

[B63] Steinhoff B. J. Schuler M. Mighali M. Intravooth T. (2024). Critical flicker fusion in patients with epilepsy under antiseizure medication. Epileptic. Disord. 26 (2), 181–187. 10.1002/epd2.20193 38116676

[B64] Street J. S. Qiu Y. C. Lignani G. (2023). Are genetic therapies for epilepsy ready for the clinic? Epilepsy. Curr. 23 (4), 245–250. 10.1177/15357597231176234 PMC10470096 37662470

[B65] Sun Z. Q. Meng F. H. Tu L. X. Sun L. (2019). Myricetin attenuates the severity of seizures and neuroapoptosis in pentylenetetrazole kindled mice by regulating the of BDNF-TrkB signaling pathway and modulating matrix metalloproteinase-9 and GABA(A). Exp. Ther. Med. 17 (4), 3083–3091. 10.3892/etm.2019.7282 30906480 PMC6425265

[B66] Symonds J. D. Zuberi S. M. Stewart K. McLellan A. O'Regan M. MacLeod S. (2019). Incidence and phenotypes of childhood-onset genetic epilepsies: a prospective population-based national cohort. Brain 142, 2303–2318. 10.1093/brain/awz195 31302675 PMC6658850

[B67] Tassinari M. Mottolese N. Galvani G. Ferrara D. Gennaccaro L. Loi M. (2022). Luteolin treatment ameliorates brain development and behavioral performance in a mouse model of CDKL5 deficiency disorder. Int. J. Mol. Sci. 23 (15), 8719. 10.3390/ijms23158719 35955854 PMC9369425

[B68] Tavakoli Z. Tahmasebi Dehkordi H. Lorigooini Z. Rahimi-Madiseh M. Korani M. S. Amini-Khoei H. (2023). Anticonvulsant effect of quercetin in pentylenetetrazole (PTZ)-induced seizures in male mice: the role of anti-neuroinflammatory and anti-oxidative stress. Int. Immunopharmacol. 116, 109772. 10.1016/j.intimp.2023.109772 36731152

[B69] Terrone G. Balosso S. Pauletti A. Ravizza T. Vezzani A. (2020). Inflammation and reactive oxygen species as disease modifiers in epilepsy. Neuropharmacology 167, 107742. 10.1016/j.neuropharm.2019.107742 31421074

[B70] Thijs R. D. Surges R. O'Brien T. J. Sander J. W. (2019). Epilepsy in adults. Lancet 393 (10172), 689–701. 10.1016/S0140-6736(18)32596-0 30686584

[B71] Vezzani A. Balosso S. Ravizza T. (2019). Neuroinflammatory pathways as treatment targets and biomarkers in epilepsy. Nat. Rev. Neurol. 15 (8), 459–472. 10.1038/s41582-019-0217-x 31263255

[B72] Wu Y. Wei H. Li P. Zhao H. Li R. Yang F. (2022). Quercetin administration following hypoxia-induced neonatal brain damage attenuates later-life seizure susceptibility and anxiety-related behavior: modulating inflammatory response. Front. Pediatr. 10, 791815. 10.3389/fped.2022.791815 35223693 PMC8873174

[B73] Xiao J. (2017). Dietary flavonoid aglycones and their glycosides: which show better biological significance? Crit. Rev. Food. Sci. Nutr. 57 (9), 1874–1905. 10.1080/10408398.2015.1032400 26176651

[B74] Yang J. Jia Z. Xiao Z. Zhao J. Lu Y. Chu L. (2021). Baicalin rescues cognitive dysfunction, mitigates neurodegeneration, and exerts anti-epileptic effects through activating TLR4/MYD88/Caspase-3 pathway in rats. Drug. Des. Devel. Ther. 15, 3163–3180. 10.2147/DDDT.S314076 PMC8312624 34321866

[B75] Yang J. Wang C. Xin W. Liu J. Ping X. Lu Y. (2024). Exploring the therapeutic potential of baicalin: mitigating anxiety and depression in epileptic rats. Comb. Chem. High. Throughput. Screen 27. 10.2174/0113862073316021240520110301 38778616

[B76] Zhang Y. J. Gan R. Y. Li S. Zhou Y. Li A. N. Xu D. P. (2015). Antioxidant phytochemicals for the prevention and treatment of chronic diseases. Molecules 20 (12), 21138–21156. 10.3390/molecules201219753 26633317 PMC6331972

[B77] Zhen J. L. Chang Y. N. Qu Z. Z. Fu T. Liu J. Q. Wang W. P. (2016). Luteolin rescues pentylenetetrazole-induced cognitive impairment in epileptic rats by reducing oxidative stress and activating PKA/CREB/BDNF signaling. Epilepsy. Behav. 57 (Pt A), 177–184. 10.1016/j.yebeh.2016.02.001 26967006

[B78] Zhuang J. Peng Y. Gu C. Chen H. Lin Z. Zhou H. (2021). Wogonin accelerates hematoma clearance and improves neurological outcome via the PPAR-γ pathway after intracerebral hemorrhage. Transl. Stroke. Res. 12 (4), 660–675. 10.1007/s12975-020-00842-9 32918259

